# Assessment of the Defatting Efficacy of Mechanical and Chemical Treatment for Allograft Cancellous Bone and Its Effects on Biomechanics Properties of Bone

**DOI:** 10.1111/os.12639

**Published:** 2020-03-18

**Authors:** Kun‐chi Hua, Jiang‐tao Feng, Xiong‐gang Yang, Feng Wang, Hao Zhang, Li Yang, Hao‐ran Zhang, Ming‐you Xu, Ji‐kai Li, Rui‐qi Qiao, Deng‐xing Lun, Yong‐cheng Hu

**Affiliations:** ^1^ Department of Bone Tumor Tianjin Hospital Tianjin China; ^2^ Graduate School Tianjin Medical University Tianjin China; ^3^ Deng‐xing Lun, MD, Department of Spine Surgery, Weifang People's Hospital Weifang China

**Keywords:** Allograft, Defatting, Biomechanical, Washing, Alcohol

## Abstract

**Objective:**

To assess the defatting efficacy of high pressure washing and gradient alcohol and biomechanical properties of defatted bone.

**Methods:**

Fresh cancellous bone was obtained from the femoral condyle and divided into six groups according to different defatting treatments, which were: high pressure washing for 10 s (10S group), 20 s (20S group), and 30 s (30S group), gradient alcohol immersion (Alcohol group), acetone immersion (Acetone group), and non‐defatted (Fresh group). The appearance of six groups was observed, and the appearance of defatted bone and fresh bone was compared. The residual lipid content and infrared spectrum were used to compare the efficacy of defatting, the DNA content was used to compare the cell content after defatting, and the maximum stress and elastic modulus were used to compare the effects of defatting treatment on biomechanical properties.

**Results:**

The fresh bone was yellow and the pores contained a lot of fat. The defatted bone was white and the porous network was clear. There was no difference in residual lipid content among the three groups with high pressure washing (1.45% ± 0.16%, 1.40% ± 0.13%, and 1.46% ± 0.11%, respectively) (*P* = 0.828). There was no difference in residual lipid content among the 10S, alcohol, and acetone groups (1.45% ± 0.16%, 1.28% ± 0.07%, and 1.13% ± 0.22%, respectively) (*P* = 0.125). Infrared spectra showed that the fat content of the five defatting groups was significantly lower than that of the fresh group. There was no difference in residual lipid content among the three groups with high pressure washing (4.53 ± 0.23 ug/mL, 4.61 ± 0.18 ug/mL, and 4.66 ± 0.25 ug/mL, respectively) (*P* = 0.645). There was no difference in residual lipid content among the 10S, alcohol, and acetone groups (4.53 ± 0.23 ug/mL, 4.29 ± 0.24 ug/mL, and 4.27 ± 0.29 ug/mL, respectively) (*P* = 0.247). The maximum stress of the bone decreased significantly with the increase of the washing time (9.95 ± 0.31 Mpa, 9.07 ± 0.45 Mpa, and 8.17 ± 0.35 Mpa, respectively) (*P* = 0.003). The elastic modulus of the bone decreased significantly with the increase of the washing time (116.40 ± 3.54 Mpa, 106.10 ± 5.29 Mpa, and 95.63 ± 4.08 Mpa, respectively) (*P* = 0.003). There was no statistical difference in the maximum stress between the fresh group, the 10S group, the alcohol group, and the acetone group (10.09 ± 0.67 Mpa, 9.95 ± 0.31 Mpa, 10.11 ± 0.07 Mpa, and 10.09 ± 0.39 Mpa, respectively) (*P* = 0.963). There was no statistical difference in the maximum stress between the fresh group, the 10S group, the alcohol group and the acetone group (119.93 ± 4.94 Mpa, 116.40 ± 3.54 Mpa, 118.27 ± 0.85 Mpa, 118.10 ± 4.52 Mpa, respectively) (*P* = 0.737).

**Conclusion:**

The defatting efficiency was satisfactory at a time of 10 s under high pressure washing. In terms of defatting efficiency and its effect on biomechanical properties of bone, high pressure washing and gradient alcohol were similar to conventional acetone solvent extraction defatting.

## Introduction

The lipid component in allograft bone graft material will reduce the safety and effectiveness of the transplantation^1^, ^2^. Because intra‐bone fat and lipoproteins and liposoluble glycopeptides on cell membranes have been proven to be important antigenic components in bone transplantation, lipid components can cause immune rejection^3^, ^4^. In addition, because the fat in the bone tissue fills the pores of the bone marrow cavity and the lumen, the hydrophobicity of the fat makes it difficult for some chemical reagents to enter the pores of the bone matrix, which affects the wettability of the material. As a result, important procedures such as decellularization and deproteinization that reduce immunogenicity cannot be performed effectively^5^, ^6^. The presence of fat reduces the bone conduction capacity of allograft bone and affects its osteogenesis ability. The fat formed on the surface of bone trabecula forms a barrier to prevent cell growth. It can also cause macrophages to react and cause excessive absorption of bone tissue, resulting in a large amount of fibrous tissue at the implantation site; these will affect the osteogenic properties of the graft material^7^, ^8^. The presence of fat will increase the cytotoxicity of the transplanted material. Irradiation is a commonly used method for allograft bone sterilization. Even allograft bone obtained under sterile conditions needs to be sterilized. γ‐irradiation is a strong oxidative process. The oxidized or peroxidized lipids are generated in the bone lipid components after irradiation, and the content of peroxidized lipids in bone after irradiation is increased by two to three times compared with before irradiation^9^. The non‐defatted allograft bone is brown after irradiation and sterilization. This change is thought to be related to the massive production of lipofuscin‐like substances produced by lipid oxidation, and lipofuscin is considered to be one of the important indicators of cell aging^10^, ^11^. Therefore, defatting has become the primary procedure for obtaining allograft bone.

The defatting treatment is mainly divided into two categories: chemical and mechanical. The chemical treatment mainly uses organic solvents to extract fats. Organic solvent extraction is used to extract the lipid components in allograft bone with volatile organic solvents. This technique can use either pure organic solvents or mixtures of organic solvents. Common solvents are petroleum ether, ethanol, methanol, chloroform, dichloroethane, and acetone. Acetone, as a classic defatting solvent, has been proven to have an excellent defatting efficiency, and it is also a commonly used treatment^12^, ^13^. Mechanical treatment is mainly based on ultrasonic cleaning. The high‐frequency vibration removes fat from the surface of bone tissue and pores. Using this treatment alone cannot effectively remove fat from bone, and it is generally used in conjunction with organic solvents. In addition to the above treatments, some researchers have proposed the use of lipase to remove fat components from bone tissue through the hydrolysis of enzymes, and the effectiveness of this treatment was confirmed by degreasing the porcine bone^14^. However, there are many types of lipases, and the enzymes have different hydrolysis capabilities, which makes them difficult to use as a conventional defatting treatment. Based on the principle of extraction, researchers have proposed a treatment for defatting using supercritical fluid extraction, which uses the high permeability, high diffusivity, and high solubility of fluids in a region (supercritical region) above the critical point. The lipid component is extracted, and almost no organic solvent is used in the extraction process. There is no solvent residue in the extract and no pollution to the environment. Supercritical fluid has both gas and liquid duality. It has both high permeability and low viscosity, equivalent to gas, and similar density to and excellent dissolving power like liquid^15^, ^16^, ^17^, ^18^. However, the equipment required for supercritical fluid extraction is expensive and complicated to operate, which has become an important reason for limiting the widespread promotion of this treatment.

Gradient alcohol treatment is commonly used for dehydration and decellularization of biological samples. If a high concentration of alcohol is used during the defatting process, the intracellular proteins will rapidly denature, and denaturation will prevent alcohol from entering the cells. Using gradient alcohol with increasing concentration can avoid this phenomenon and make the solvent contact the cells more fully. Gardin *et al*.^19^ studied the defatting technology of xenogeneic bone with defatting treatment with gradient alcohol, and found that the fat content of bone treated with gradient alcohol was significantly lower than that of bone without gradient alcohol. Gradient alcohol treatment can cause fat cell rupture and lipid dissolution at the same time, to achieve the purpose of defatting. The high pressure water gun is a high pressure cleaner, commonly known as a high pressure water jet cleaner. It is a machine that uses a power unit to make the high pressure plunger pump produce high pressure water to wash the surface of the object. It can peel off dirt and wash away, and achieve the purpose of cleaning the surface of objects. High pressure washing removes lipids and blood from bone and reduces the number of bacteria in the graft. Because a high pressure water column is used to clean up the bone, there is no need to worry about the problem of chemical residues. In practical applications, no practical research has been conducted on the duration of high pressure washing and defatting efficiency. Reasonable washing time is an important parameter for high pressure washing, because bone is bound to withstand continuous washing with a high pressure water column. However, insufficient washing time will also cause a large amount of residual immunogenic substances in the bone tissue, especially fat.

The purpose of the present study was to (i) find a reasonable high pressure washing time that would ensure effective removal of fat and unaffected mechanical properties of bone tissue; (ii) evaluate the effects of acetone, gradient alcohol, and high pressure washing on the defatting efficiency and biomechanical properties of allograft cancellous bone.

## Materials and Methods

### *Allograft Bone*

The method for obtaining human cancellous bone is as follows: (i) place fresh human femoral condyle into a sterile plastic bag and freeze it at −80°C for more than 4 weeks; (ii) obtain 5 × 5 × 5 mm cancellous bone; and (iii) the obtained cancellous bone pieces are put into deionized water and washed with an ultrasonic cleaner for 4 h, and the deionized water is replaced every hour. After processing, the bone pieces were dried in a 50°C drying box for 6 h. After drying, the sample polyethylene was vacuum‐sealed.

### *Grouping*

The prepared bone pieces were divided into six groups, and five groups were selected for defatting treatment. The mechanical treatment was performed by high pressure washing. The washing time was 10 s (10S group), 20 s (20S group), and 30 s (30S group). Chemical treatment used the gradient alcohol method (alcohol group). The standard control group was treated with acetone (acetone group). The remaining one group of non‐defatted bone was used as a control (fresh group).

### *Defatting Process*

Processing with high pressure washing: bone cubes were placed in a self‐made nylon net bag, the opening of the net bag was tightened, the spray gun head was extended into the net bag, and the tip of the gun was positioned 10 cm away from the surface of the bone. In the process of detachment, we chose a columnar spray head, set the cleaning pressure to 6 MPa, with voltage 220 V/50 Hz, power 1.6 kw, and speed 2800 r/min, before washing for 10 s (10S group), 20 s (20S group), and 30 s (30S group).

Processing with gradient alcohol (Alcohol group): first, we used deionized water and 100% ethanol to make 50% alcohol. We placed the bone cubes in a 200‐mL beaker; we added 150 mL 50% alcohol, soaking for 2 h, then soaked the bone cubes in 150 mL 75% alcohol for 2 h, then 150 mL 95% alcohol for 2 h, and, finally, in 150 mL of 100% ethanol for 2 h. During the process, the mechanical stirrer was used for stirring, and the rotation speed was set to 200 r/min.

Processing with acetone (Acetone group): first, deionized water and 100% ethanol were used to make 50% alcohol. We placed the bone cubes in a 200‐mL beaker and added 150 mL of acetone, soaking for 3 h; we than soaked the bone cubes in 150 mL of 50% alcohol for 1 h, then 150 mL of acetone for 3 h and, finally, in 150 mL of 50% alcohol for 1 h. In the process, the mechanical stirrer was used for stirring, and the rotation speed was set to 200 r/min.

Fresh group: Bone pieces were placed in a 200‐mL beaker, 150 mL of deionized water was added, and the deionized water was replaced every 2 h. During the process, the mechanical stirrer was used for stirring, and the speed was set to 200 r/min.

After the six groups were finished, they were placed into deionized water and washed with ultrasonic cleaner for 12 h, with the deionized water replaced every 3 h. The washed bone pieces were dried in a 50°C dry oven for 6 h. After drying, the bone pieces were placed in a desiccator at room temperature. After being sealed, they were placed in a −20°C refrigerator and stored frozen (Table 1).

**Table 1:** The main materials and equipment companies, model, and important parameters

Material and equipment	Company	Model	Parameter
Deionized water	Wonderful Biological Materials, Beijing, China	—	—
Alcohol	Beijing Chemical Reagent Company, Beijing, China	75%, 95%, and 100% v/v	—
Acetone	Tianjin Damao Chemical Reagent Factory, Tianjin, China	Analytical pure	—
Petroleum ether	Tianjin Damao Chemical Reagent Factory, Tianjin, China	30°C–60°C	—
Animal Tissue/Cell Genomic DNA Extraction Kit	Solarbio, Beijing, China	D1700–100T	Store dry at room temperature (15−25°C), retest period is 12 months
Potassium Bromide powder	Bangjing Industrial, Shanghai, China	Spectral purity	—
−80°C refrigerator	Zhongke Meiling Cryogenic Technology, Anhui, China	DW‐HL828	Temperature inside the box: −10°C–−86°C; effective volume: 828 L; rated voltage: 220 V
−20°C refrigerator	Hefei Midea Refrigerator, Anhui, China	BD/BC‐96KM(E)	Freezing temperature in the box: −16°C–−24°C; freezing capacity: 16 kg/24 h; structural features: flip‐top door, can stay at any angle within ≥30° and ≤75°
High pressure Rinsing Gun	Jeremy Equipment, China	GTQ‐1600	Cylindrical nozzle, cleaning pressure 6 MPa; fan nozzle, cleaning pressure 4–5 MPa
Ultrasonic cleaner	Kexi Century Technology, Beijing, China	KX‐1024	Ultrasonic power 1200 W, ultrasonic frequency: 28 KHz, heating power 2000 W
Digital display constant speed electric mixer	Changzhou Ronghua Instrument Manufacturing, Jiangsu, China.	JJ‐1H	—
High‐speed pulverizer	Red Sun Electromechanical, Zhengjiang, China	RRH‐A1000	—
Soxhlet extractor	Shubo Glass Instrument, Sichuan, China	250 mL	—
Constant Temperature Water bath	Guohua Electric, Jiangsu, China	HH‐2	—
Fourier transform Infrared Spectrometer	Thermo Fisher Scientific, Massachusetts, Usa	Nicolet iS10	Spectral resolution: better than 0.4 cm^−1^; spectral range: 7800–350 cm^−1^; wave number accuracy: 0.01 cm^−1^
Desktop High‐speed micro Mini centrifuge	Solarbio, Beijing, China	YZ‐D2012 plus	The maximum speed is 15 000 rpm, and the maximum relative centrifugal force (RCF) is 15 100 *g*
Uv spectrophotometer	Inesa Analytical Instrument, Shanghai, China	L5S	Transmittance measurement range: 0.0% −200.0%; absorbance measurement range: 0.301A–4.000A
Electronic universal testing machine	Jinan Chuanbai Instrument Equipment, Shandong, China	WDW‐10	The maximum test force is 10 kN, the relative error of the test force is ±1%, the effective measurement force range is 0.2%–100% FS, and the beam moving speed is 0.001 to 500 mm/min
Electronic balance	Jingqi Instrument, Shanghai, China		Weighing range is 0–200 g, standard deviation is 0.0002 g

### *Appearance*

We observed the general shape of the bone and the morphology and color of the material. The clearer the pores of the bone and the whiter the color, the more effective the treatment proved.

### *Residual Lipid Content*

Soxhlet extraction was used to determine the residual lipid content of bone mass after defatting. Soxhlet extraction was a method of extracting compounds from solid matter. Five groups of bone pieces were pulverized to bone particles with a particle size of <900 μm using a high‐speed pulverizer. The qualitative filter paper was folded into a filter paper bag, and the analytical balance was precisely weighed. Each group of bone particles was equally divided into five parts. After being put into the filter paper bag, the weight was precisely weighed again to obtain the weight of each bone particle, which was recorded as M_1_. The filter paper bag was placed into the siphon, and 150 mL of petroleum ether was added. The Soxhlet extractor was connected in sequence, and the connected instrument was put into a thermostatic water bath, setting the water temperature to 50°C, starting timing with the first siphon, continuing extracting for 24 h, and keeping petroleum ether to rinse the sample every 10 minutes. After Soxhlet extraction was completed, the filter paper bag was taken out, dried at 50°C for 6 h, and placed in a desiccator at room temperature. We precisely weighed the total weight of the dried filter paper bag and the weight of the filter paper, calculated the bone after defatting, and recorded it as M_2_. Residual lipid content calculation formula:

Residual lipid content = (M_1_ − M_2_)/M_1_ × 100%.

Note: M_1_ was the mass before Soxhlet extraction, and M_2_ was the mass after Soxhlet extraction.

The residual lipid content was a continuous variable. The lower the value, the better the efficacy of the defatting treatment.

### *Infrared Spectrometer*

In this experiment, infrared spectroscopy was used to observe the changes of lipid components in bone after different defatting treatments, and comparison was made with fresh bone. Infrared spectroscopy was a method used for structural analysis based on the selective absorption of electromagnetic radiation in the infrared region by different substances, as well as for analyzing the composition of various compounds that absorb infrared light. We took 2 mg of bone from each of the six groups for grinding, then added approximately 200 mg of pure KBr powder for even grinding, before placing into a mold and pressing (110 Pa pressure) on a hydraulic press to form a transparent sheet. After the preparation was completed, the potassium bromide tablet containing the sample was placed on a magnetic tablet holder, which was then, together with the tablet holder, placed in the optical path of the infrared spectrometer, and scanned in the range of 4000–400 cm^−1^ to draw an infrared absorption spectrum. Compared with the infrared spectrum of fresh bone, the lower the characteristic absorption peak of the lipid component, the higher the efficiency of the defatting treatment.

### *DNA Content*

**Figure 1:**
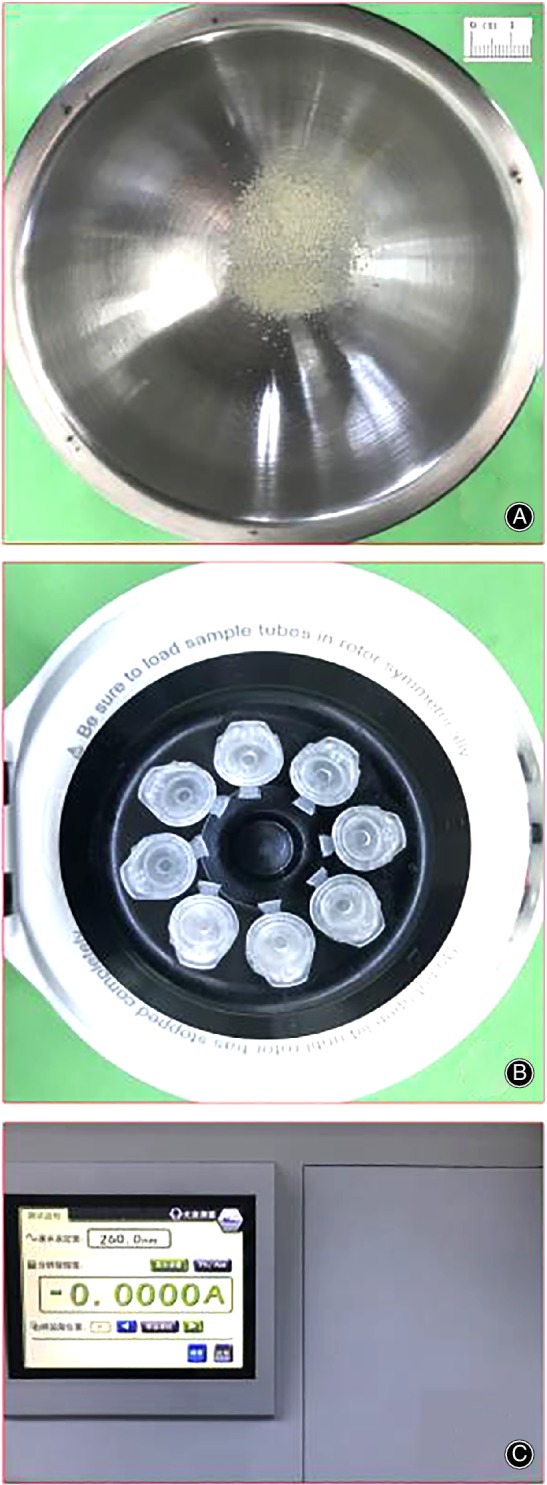
DNA content determination process: grinding bone mass (A), centrifugation (B), and UV spectrophotometer to measure OD (optical density) value (C). Bone mass was ground to bone powder with a diameter of 0.80–1.50 mm. When centrifuging, we put the balanced centrifuge tube symmetrically into the centrifugal rotor (position balance), covered the centrifugal rotor, and paid attention to whether it was tight. The wavelength setting value of the ultraviolet spectrophotometer was 260.0 nm, and it was adjusted to “zero” after the setting was completed.

DNA content could represent the amount of cells remaining in the bone. In this experiment, the DNA content in each group of bone was measured to compare the difference in the remaining cells in different defatted bone. The animal tissue/cell genomic DNA extraction kit was used to isolate the DNA from the bone. After obtaining high‐quality genomic DNA, an ultraviolet spectrophotometer (wavelength setting is 260 nm) was used to obtain the optical density (OD) value of each group solution and we calculated the corresponding DNA content (Fig. 1).

DNA content (ug/mL) = OD value × 50 ug/mL × dilution factor.

Each group of tests was repeated five times. DNA content was a continuous variable. The lower the value, the lower the number of remaining cells in the group.

### *Biomechanical Test*

Biomechanics refers here to the use of an electronic universal testing machine to detect the biomechanical properties of bone. This experiment mainly observed the maximum stress and elastic modulus of the bone. The maximum stress refers to the reaction force generated per unit area when the material was about to be damaged by external force, which was the limit value for the material to work safely. The elastic modulus refers to the stress required for a unit to deform elastically under the action of an external force, and it is an index reflecting the material's ability to resist elastic deformation. Six bone blocks were taken from each group for biomechanical testing. Before the test, we ensured that the force line of the loading device was perpendicular to the surface of the bone block and performed a displacement control loading (1 mm/min) biomechanical test. The ambient temperature was 20°C and the humidity was 50%. The test was stopped when the bone tissue was deformed or ruptured to obtain the maximum stress and elastic modulus. The maximum stress and elastic modulus were continuous variables. The larger the value, the better the biomechanical properties of the defatted bone.

### *Statistical Analysis*

Statistical analysis was processed with SPSS 20.0 (Statistical Package for Social Sciences, IBM, USA) statistical software. Continuous variables (residual lipid content, DNA content, maximum stress, and elastic modulus) conforming to the normal distribution were expressed as mean ± standard deviation (x ± s). One‐way ANOVA was used for comparison between multiple groups. For further pairwise comparisons, the SNK *q*‐test was used for all variances, and the LSD *t*‐test was used for variances. For the two‐sided test, when *P* < 0.05, the difference was considered statistically significant.

## Results

### *Appearance Results*

**Figure 2:**
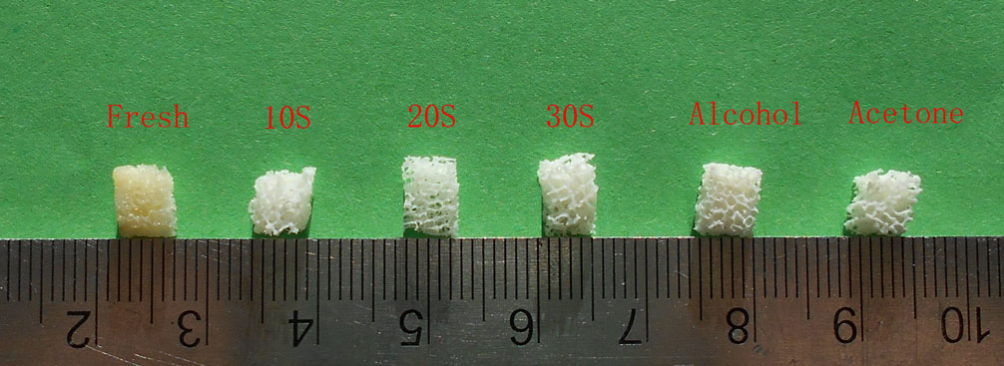
Appearance of fresh group and five groups defatted bone. From left to right, fresh group, 10S group, 20S group, 30S group, alcohol group, and acetone group. The fresh bone was yellow with a lot of fat in the pores, and the defatted bone mass was white and the porous network structure was clear.

The surface of fresh bone mass was rough and covered with fat, and the pores were not obvious. The defatted bone was white and had a clear porous structure. The pores communicated with each other, and the pore walls were clean without soft tissue adhesion (Fig. 2).

### *Measurement of Residual Lipid Content*

**Figure 3:**
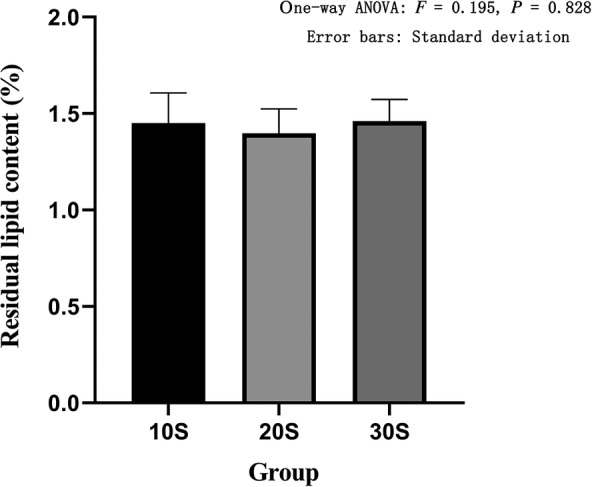
Residual lipid content in three groups of high pressure washing. The abscissa represents the grouping and the ordinate represents the residual lipid content. Error bars represent standard deviations. One‐way ANOVA was used to compare the residual lipid content between the three groups (*F* = 0.195, *P* = 0.828). It was proved that there was no statistical difference in the residual lipid content among the three groups.

**Figure 4:**
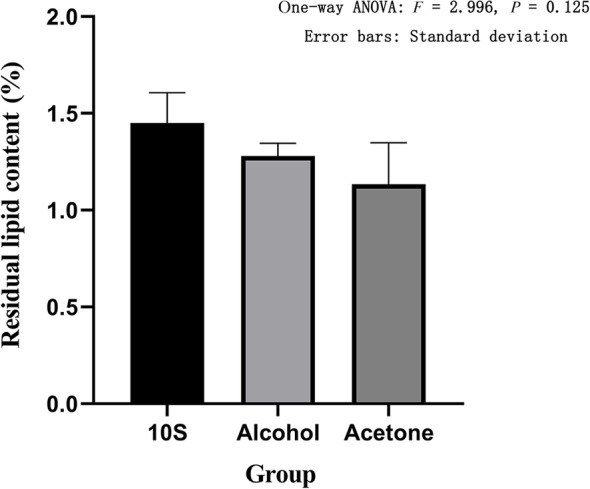
Residual lipid content in different defatting treatments. The abscissa represents the grouping and the ordinate represents the residual lipid content. Error bars represent standard deviations. One‐way ANOVA was used to compare the residual lipid content between the three groups (*F* = 2.966, *P* = 0.125). It was proved that there was no statistical difference in the residual lipid content among the three groups.

The Soxhlet extraction method was used to determine the residual lipid content of each group. The residual lipid content of the 10S group was 1.45% ± 0.16%, the residual lipid content of the 20S group was 1.40% ± 0.13%, the residual lipid content of the 30S group was 1.46% ± 0.11%, the residual lipid content in the alcohol group was 1.28% ± 0.07%, and the residual lipid content in the acetone group was 1.13% ± 0.22%. The 10S, 20S, and 30S groups showed no statistical difference between the three groups based on one‐way ANOVA (*F* = 0.195, *P* = 0.828) (Fig. 3). The residual fat content of the 10S group, the alcohol group, and the acetone group showed that there was no statistical difference between the three groups based on one‐way ANOVA (*F* = 2.996, *P* = 0.125) (Fig. 4).

### *Measurement Results of Infrared Spectrometer*

**Figure 5:**
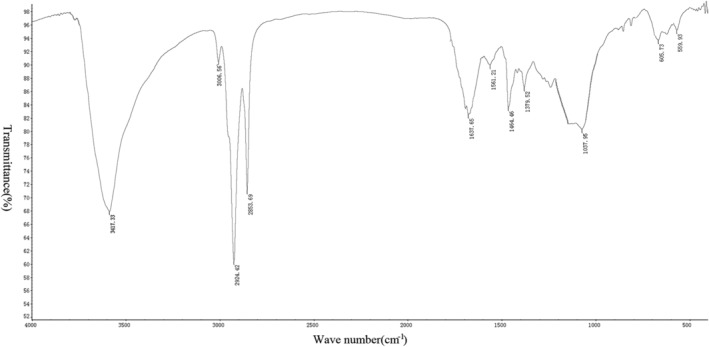
Infrared spectrum of fresh group: the small absorption peaks at 559 cm^−1^ and 605 cm^−1^ were caused by the asymmetric bending vibration of the O─P─O bond in PO_4_^3−^; the strong absorption peak at 1037 cm^−1^ was the asymmetric stretching vibration peak of the P─O bond in PO_4_^3−^. Asymmetric stretching vibration of CO_3_^2−^ could be observed at 1464 and 1561 cm^−1^, indicating that the bone contains more carbonate. The bending vibration peak of hydroxyl groups in water appeared at 1637 cm^−1^, which indicated that the bone still contained a certain amount of moisture, while the strong absorption peak generated by hydroxyl groups in hydroxyapatite appeared at 3417 cm^−1^. There were two strong absorption peaks at 2853 and 2924 cm^−1^, which were mainly formed by the C‐H stretching vibration in saturated fatty acids, which proved that the fresh group bone contains a lot of fat.

**Figure 6:**
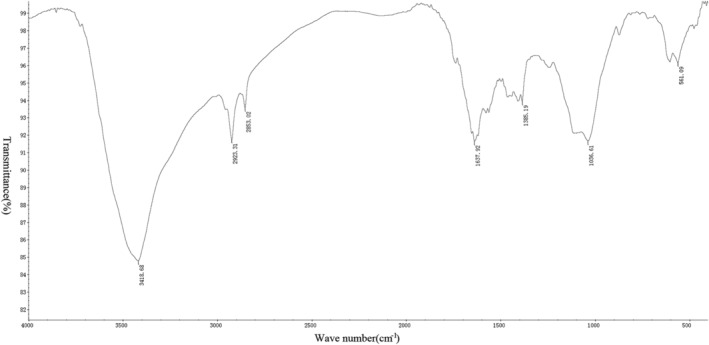
Infrared spectrum of 10S group: the small absorption peak at 561 cm^−1^ was caused by the asymmetric bending vibration of the O─P─O bond in PO_4_^3−^. The strong absorption peak at 1036 cm^−1^ was the asymmetric stretching vibration peak of the P─O bond in PO_4_^3−^. Asymmetric stretching vibration of CO_3_^2−^ could be observed at 1385 cm^−1^, indicating that the bone contains carbonate. The bending vibration peak of hydroxyl groups in water appeared at 1637 cm^−1^, which indicated that the bone still contained a certain amount of moisture, while the strong absorption peak generated by hydroxyl groups in hydroxyapatite appeared at 3418 cm^−1^. There were two absorption peaks at 2853 and 2923 cm^−1^, which were mainly formed by the C‐H stretching vibration in saturated fatty acids, which proved that the bone in the 10S group still contains a small amount of fat.

**Figure 7:**
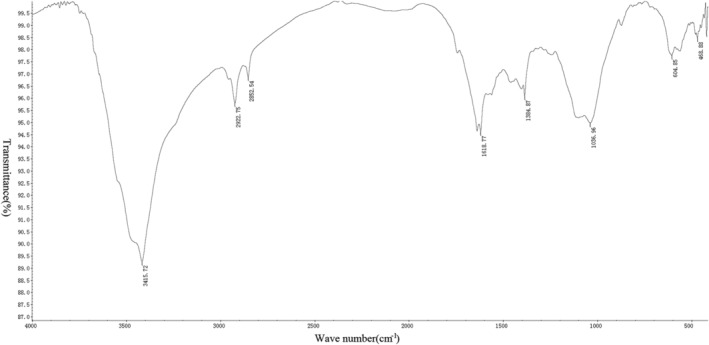
Infrared spectrum of 20S group: the small absorption peak at 604 cm^−1^ was caused by the asymmetric bending vibration of the O─P─O bond in PO_4_^3−^. The strong absorption peak at 1036 cm^−1^ was the asymmetric stretching vibration peak of the P─O bond in PO_4_^3−^. Asymmetric stretching vibration of CO_3_^2−^ could be observed at 1384 cm^−1^, indicating that the bone contains carbonate. A bending vibration peak of hydroxyl groups in water appeared at 1618 cm^−1^, indicating that bone still contained a certain amount of moisture, while a strong absorption peak generated by hydroxyl groups in hydroxyapatite appeared at 3415 cm^−1^. There were two absorption peaks at 2852 and 2922 cm^−1^, which were mainly formed by the C‐H stretching vibration in saturated fatty acids, which proved that the bone in the 20S group still contains a small amount of fat.

**Figure 8:**
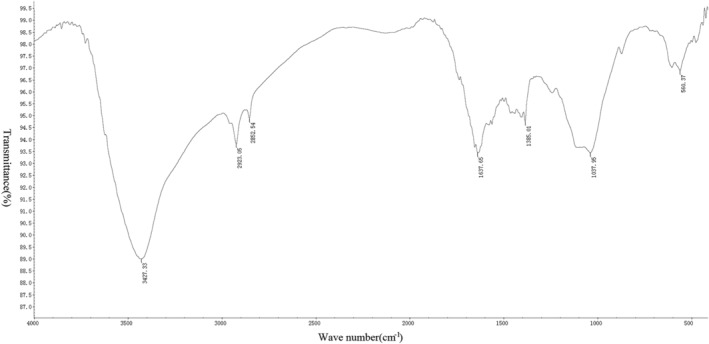
Infrared spectrum of 30S group: the small absorption peak at 560 cm^−1^ was caused by the asymmetric bending vibration of the O─P─O bond in PO_4_^3−^. The strong absorption peak at 1037 cm^−1^ was the asymmetric stretching vibration peak of the P─O bond in PO_4_^3−^. Asymmetric stretching vibration of CO_3_^2−^ could be observed at 1385 cm^−1^, indicating that the bone contains carbonate. The bending vibration peak of hydroxyl groups in water appeared at 1637 cm^−1^, indicating that the bone still contained a certain amount of water, while the strong absorption peaks of hydroxyl groups in hydroxylapatite appeared at 3427 cm^−1^. There were two absorption peaks at 2852 and 2923 cm^−1^, which were mainly formed by the C‐H stretching vibration in saturated fatty acids, which proved that the bone in the 30S group still contains a small amount of fat.

**Figure 9:**
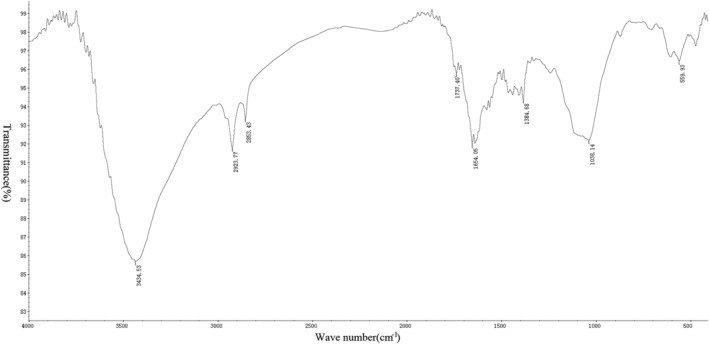
Infrared spectrum of alcohol group: the small absorption peak at 559 cm^−1^ was caused by the asymmetric bending vibration of the O─P─O bond in PO_4_^3−^. The strong absorption peak at 1038 cm^−1^ was the asymmetric stretching vibration peak of the P─O bond in PO_4_^3−^. Asymmetric stretching vibration of CO_3_^2−^ could be observed at 1384 cm^−1^, indicating that the bone contains carbonate. The bending vibration peak of hydroxyl groups in water appeared at 1654 cm^−1^, indicating that the bone still contained a certain amount of moisture, while the strong absorption peaks of hydroxyl groups in hydroxyapatite appeared at 3434 cm^−1^. There were two absorption peaks at 2853 and 2923cm^−1^, which were mainly formed by the C─H stretching vibration in saturated fatty acids, which proved that the bone in the alcohol group still contains a small amount of fat.

**Figure 10:**
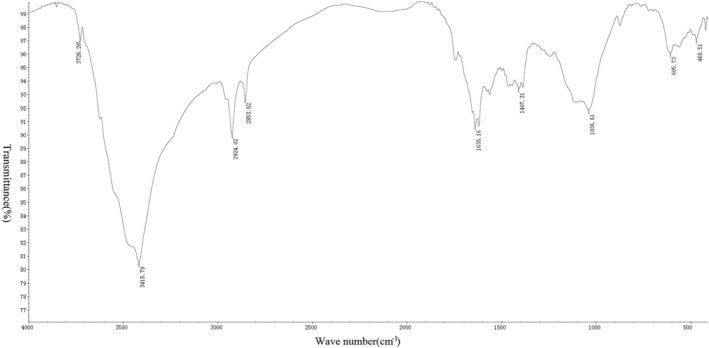
Infrared spectrum of acetone group: the small absorption peak at 605 cm^−1^ was caused by the asymmetric bending vibration of the O─P─O bond in PO_4_^3−^. The strong absorption peak at 1036 cm^−1^ was the asymmetric stretching vibration peak of the P─O bond in PO_4_^3−^. Asymmetric stretching vibration of CO_3_^2−^ could be observed at 1407 cm^−1^, indicating that the bone contains carbonate. The bending vibration peak of hydroxyl groups in water appeared at 1638 cm^−1^, indicating that the bone still contained a certain amount of moisture, while the strong absorption peak generated by hydroxyl groups in hydroxyapatite appeared at 3415 cm^−1^. There were two absorption peaks at 2853 and 2924 cm^−1^, which were mainly formed by the C‐H stretching vibration in saturated fatty acids, which proved that the bone in the acetone group still contains a small amount of fat.

After the tablet preparation was completed, six groups of spectra were drawn using an infrared spectrometer (Figs 5, 6, 7, 8, 9, 10). Compared with the fresh group, at 2916–2936 cm^−1^, the absorption peak height of the five groups of defatted bone mass was significantly reduced, and this peak was formed by the stretching vibration of C‐H in saturated fat, which represented fat content; the fat content of defatted bone mass was significantly reduced. In addition, the absorption peaks related to OH^−^, H^+^, PO_4_^3−^, and CO_3_^2−^ in the five defatting groups had almost no difference in position and intensity compared with fresh bone mass, indicating that these defatting treatments could maintain the basic composition and the natural structural state.

### *Measurement of DNA Content*

**Figure 11:**
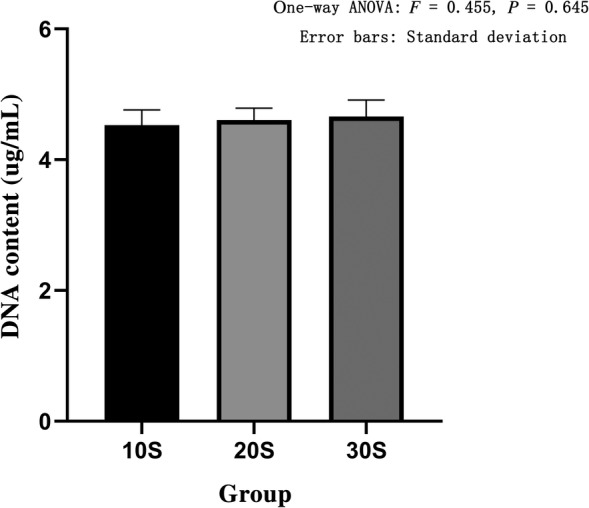
DNA content in three groups of high pressure washing. The abscissa represents the grouping and the ordinate represents the DNA content. Error bars represent standard deviations. One‐way ANOVA was used to compare the DNA content between the three groups (*F* = 0.455, *P* = 0.645). It was proved that there was no statistical difference in the DNA content among the three groups.

**Figure 12:**
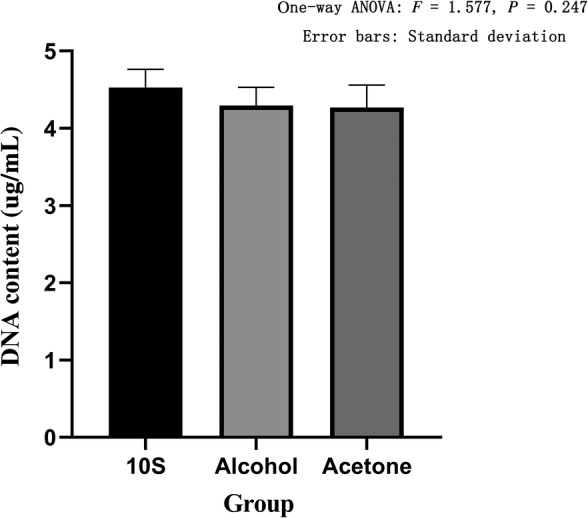
DNA content in different defatting treatments. The abscissa represents the grouping and the ordinate represents the DNA content. Error bars represent standard deviations. One‐way ANOVA was used to compare the DNA content between the three groups (*F* = 1.577, *P* = 0.247). It was proved that there was no statistical difference in the DNA content among the three groups.

We used an UV spectrophotometer to obtain the OD value, and then calculated the DNA content of each group. The DNA content of the 10S group was 4.53 ± 0.23 ug/mL, the DNA content of the 20S group was 4.61 ± 0.18 ug/mL, the DNA content of the 30S group was 4.66 ± 0.25 ug/mL, the DNA content of the alcohol group was 4.29 ± 0.24 ug/mL, and the DNA content of the acetone group was 4.27 ± 0.29 ug/mL. The 10S, 20S, and 30S groups showed no statistical difference between the three groups based on one‐way ANOVA (*F* = 0.455, *P* = 0.645) (Fig. 11). The DNA content of the 10S, the alcohol, and the acetone groups showed that there was no statistical difference between the three groups based on one‐way ANOVA (*F* = 1.577, *P* = 0.247) (Fig. 12).

### *Results of Biomechanical Tests*

**Figure 13:**
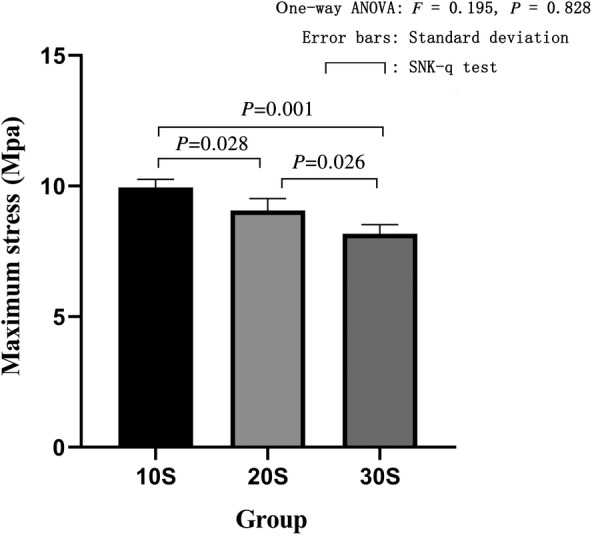
Maximum stress in three groups of high pressure washing. The abscissa represents the grouping and the ordinate represents the maximum stress. Error bars represent standard deviations. One‐way ANOVA was used to compare the maximum stress between the three groups (*F* = 16.96, *P* = 0.003). It was proved that there was a statistical difference in the maximum stress among the three groups. Based on further pairwise comparison, there was a statistical difference between the 10S group and the 20S group, and the 10S group was significantly higher than the 20S group. The 10S group was significantly different from the 30S group, and the 10S group was significantly higher than the 30S group. The 20S group was significantly different from the 30S group, and the 20S group was significantly higher than the 30S group.

**Figure 14:**
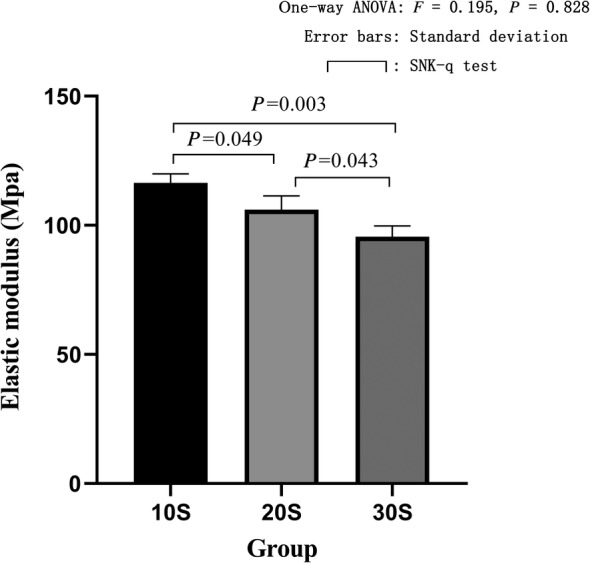
Elastic modulus in three groups of high pressure washing. The abscissa represents the grouping and the ordinate represents the elastic modulus. Error bars represent standard deviations. One‐way ANOVA was used to compare the elastic modulus between the three groups (*F* = 16.98, *P* = 0.003). It was proved that there was a statistical difference in the elastic modulus among the three groups. Based on further pairwise comparison, there was a statistical difference between the 10S group and the 20S group, and the 10S group was significantly higher than the 20S group. The 10S group was significantly different from the 30S group, and the 10S group was significantly higher than the 30S group. The 20S group was significantly different from the 30S group, and the 20S group was significantly higher than the 30S group.

**Figure 15:**
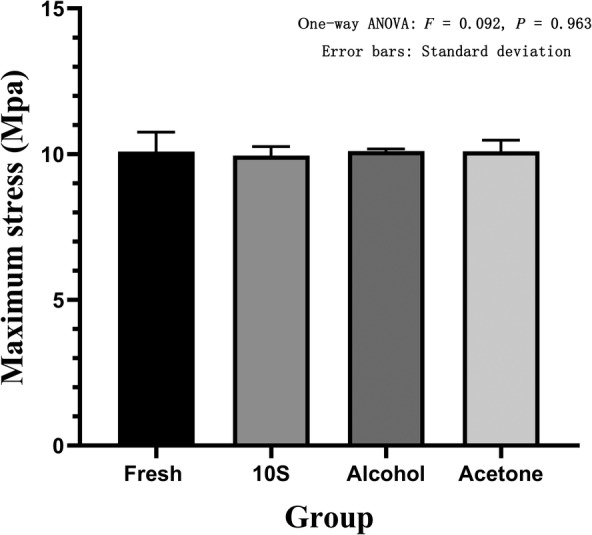
Maximum stress in fresh, 10S, alcohol, and acetone groups. The abscissa represents the grouping and the ordinate represents the maximum stress. Error bars represent standard deviations. One‐way ANOVA was used to compare the maximum stress between the four groups (*F* = 0.092, *P* = 0.963).

**Figure 16:**
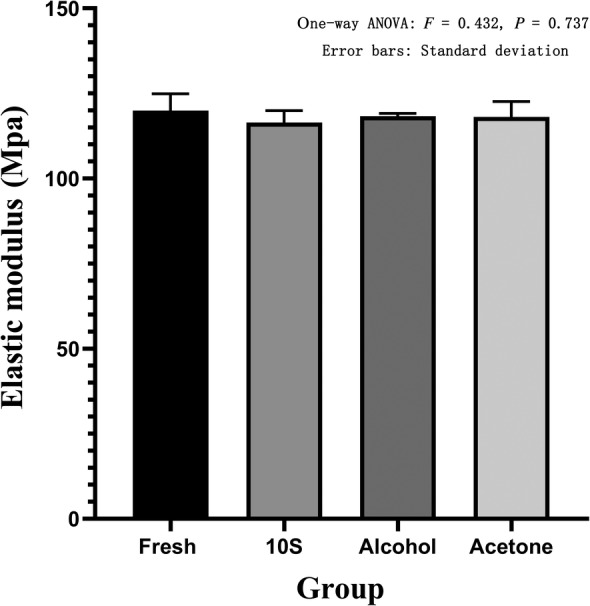
Elastic modulus in fresh, 10S, alcohol, and acetone groups. The abscissa represents the grouping and the ordinate represents the elastic modulus. Error bars represent standard deviations. One‐way ANOVA was used to compare the elastic modulus between the four groups (*F* = 0.431, *P* = 0.737).

The electronic universal testing machine performed compression failure experiments to obtain the maximum stress and elastic modulus of each group. The maximum stress of the 10S group was 9.95 ± 0.31 Mpa, and the elastic modulus was 116.40 ± 3.54 Mpa. The maximum stress of the 20S group was 9.07 ± 0.45 Mpa, and the elastic modulus was 106.10 ± 5.29 Mpa. The maximum stress of the 30S group was 8.17 ± 0.35 Mpa, and the elastic modulus was 95.63 ± 4.08 Mpa. The maximum stress of the alcohol group was 10.11 ± 0.07 Mpa, and the elastic modulus was 118.27 ± 0.85 Mpa. The maximum stress of the acetone group was 10.09 ± 0.39 Mpa, and the elastic modulus was 118.10 ± 4.52 Mpa. The maximum stress of the fresh group was 10.09 ± 0.67 Mpa, and the elastic modulus was 119.93 ± 4.94 Mpa. The one‐way ANOVA of the maximum stress (*F* = 16.96, *P* = 0.003) and the elastic modulus (*F* = 16.98, *P* = 0.003) of the three groups of high pressure washing showed statistical differences. In further pairwise comparison, in terms of maximum stress, there was a statistical difference between the 10S group and the 20S group; the 10S group was significantly higher than the 20S group (*P* = 0.028). The 10S group was significantly different from the 30S group, and the 10S group was significantly higher than the 30S group (*P* = 0.001). The 20S group was significantly different from the 30S group, and the 20S group was significantly higher than the 30S group (*P* = 0.026) (Fig. 13). In terms of elastic modulus, there was a statistical difference between the 10S group and the 20S group, and the 10S group was significantly higher than the 20S group (*P* = 0.049). There was a statistical difference between the 10S group and 30S group, and the 10S group was significantly higher than the 30S group (*P* = 0.003). The 20S group was significantly different from the 30S group, and the 20S group was significantly higher than the 30S group (*P* = 0.043) (Fig. 14). The one‐way ANOVA of the maximum stress and elastic modulus of the fresh group, 10S group, alcohol group, and acetone group revealed no statistical differences (*F* = 0.092, *P* = 0.963) (Fig. 15). The one‐way ANOVA of the elastic modulus and the elastic modulus of the fresh group showed that the 10S group, the alcohol group, and the acetone group had no statistical differences (*F* = 0.431, *P* = 0.737) (Fig. 16).

## Discussion

Resolving immune rejection among different individuals is critical for allograft transplantation. Defatting is an important treatment for reducing the immunogenicity of allograft bone^20^, ^21^. In bone transplantation, the response of the recipient to the allograft bone is mainly a T lymphocyte‐mediated response to the allograft bone cell surface antigen. T cells recognize antigens on the cell surface, not on minerals or bone matrix^22^, ^23^, ^24^. Cell surface antigens are glycoproteins, glycolipids, and glycopeptides embedded on the surface of cell membranes. They are distinguishing marks for cells and sites for immune recognition. To reduce the immunogenicity of allograft bone, it is necessary to reduce cell surface antigens or to change its structure. By removing lipoproteins and liposoluble glycopeptides from bone fat and cell membranes, the integrity of cellular antigens is destroyed, thereby reducing the immunogenicity of allograft bone^25^, ^26^, ^27^. Chappard *et al*.^7^ implanted a non‐defatted bone mass into a New Zealand rabbit. Shortly after implantation, a wide range of non‐specific inflammation was observed around the graft, and significant fibrosis appeared between the trabecular bone and newly generated giant cells. The appearance of these giant cells was similar to the appearance of Langerhans giant cells observed around the acute pancreatitis lesions, and the pathological characteristics of acute pancreatitis were mainly the release of lipase from the pancreas into the blood and enzymatic hydrolysis of adipose tissue.

The fat contained in bone tissue fills the pores of the bone marrow cavity and other lumens. Thoren *et al*.^8^, ^28^ observed the immunogenicity and osteogenic capacity of the grafts after defatting the frozen allogeneic bone and implanted them into rabbits. The results showed that defatting could remove these fats, not only increasing the wettability of allogeneic bone but also leaving pores for new bone formation. One week after the defatted allogeneic bone was implanted into the animal, it was observed that bone marrow mesenchymal stem cells grew into the void left by the lipid clearance, and new bone was also generated in small amounts. Necrosis of surrounding tissues occurred after implantation, and inflammatory cells appeared in large numbers and were wrapped around the necrotic tissue. This study confirmed that the presence of fat reduces the bone conduction capacity of allogeneic bone and affected its osteogenesis ability. The fat formed on the surface of bone trabecula creates a barrier to prevent cell growth, and could also cause macrophage responses to lead to excessive absorption of bone to form fibrous tissue filling. The bone graft material with a high degree of fat removal was easily infiltrated by body fluids after implantation in the body, and the blood vessels and tissues around the host bone were more likely to grow in, which was conducive to new bone formation.

Irradiation is a commonly used method for sterilization of allogeneic bone. Even allogeneic bone obtained under aseptic conditions needs to be sterilized by irradiation before being stored in a bone bank^29^, ^30^. Gamma irradiation is a strong oxidative process. After exposure to lipid components in bone, oxidized or peroxidized lipids are formed. These have strong cytotoxicity^31^, ^32^, ^33^. Moreau *et al*.^34^ co‐cultured irradiated human cancellous bone with osteoblasts, and the results showed that a large number of dead osteoblasts appeared around the graft, but no obvious cell death occurred around the unirradiated graft. The content of peroxidized lipid in the grafts after irradiation was significantly higher than that in the non‐irradiated group. Oxidized lipids were considered to be the main cause of the large number of osteoblastic deaths. Therefore, when preparing allogeneic bone, defatting treatment should be added to reduce the cytotoxicity of the graft.

The past defatting approaches mainly used mechanical and chemical treatments. Mechanical defatting was primarily based on ultrasonic cleaning, but the combination of ultrasonic and deionized water alone could not achieve the goal of degreasing. The chemical treatment was mainly chloroform and acetone. The organic solvent was used to extract the lipid components to achieve the goal of separating bone tissue and lipid. However, the residue of organic solvents and poor wettability of bone were important factors affecting the defatting efficacy^35^. Two novel defatting treatments were used in this experiment. One was the mechanical treatment of high pressure water gun washing, and the other was the chemical treatment of gradient alcohol soaking. In our experiments, the first issue we resolved was the determination of the time of high pressure washing. Before the formal test, we carried out a pre‐test to determine the washing time. It was found that when the washing time was greater than 30 s, the structure of bone tissue was severely damaged and could not receive further defatting treatment. Therefore, the upper limit of the washing time was determined to be 30 s. Combined with experience in actual production, the high pressure washing time was determined as 10 s (10S group), 20 s (20S group), and 30 s (30S group). In appearance, there was no significant difference in color and morphology, and the three groups were all white with clear pores. The Soxhlet extraction method was used to determine the residual lipid content of the bone mass in each group. As a result, there was no statistical difference in the residual lipid content of the three groups of bone mass. Further observation of the infrared spectrum confirmed that there was no significant difference in the remaining fat content of the three groups of bone. In addition, from the infrared spectrum, the absorption peaks of the three groups of OH^−^, H^+^, PO_4_^3−^, and CO_3_^2−^ were compared with the fresh group, and there was almost no difference in position and intensity. It showed that the treatment of high pressure washing for 10 s, 20 s, and 30 s could maintain the basic composition and the natural structural state of collagen and inorganic matter. In addition, we observed the effect of high pressure washing on the DNA content, and the results showed that there was no significant difference in the DNA content of the three groups, proving that high pressure washing for 10 s, 20 s, and 30 s had the same ability to remove cells in bone. To observe whether the prolonged time of high‐pressure washing would reduce the mechanical properties of bone, we performed biomechanical tests on three groups, mainly to observe the changes in their maximum stress and elastic modulus. The results showed that there were significant differences between the maximum stress and elastic modulus of the three groups, and the maximum stress and elastic modulus of the bone decreased significantly with the increase of the washing time. Therefore, we believed that the time of high‐pressure washing should be selected as 10 s, because prolonging the washing time could not only improve the degreasing effect but also reduce the mechanical properties of the bone.

This experiment used acetone (Acetone group) as the standard defatting scheme, and compared the efficacy of high pressure washing (10S group) and gradient alcohol (Alcohol group) treatments. Appearance observation showed that the fresh bone was rough on the surface, covered with fat, and the pores were not obvious. The defatted bone was white and clear, with a clear porous structure. The Soxhlet extraction method was used to determine the residual lipid content of the three groups of defatted bone. As a result, there was no statistical difference in the residual lipid content of the three groups. Further observation of the infrared spectrum confirmed once again the results of no significant difference in the residual lipid content of the three groups. Moreover, from the infrared spectrum, the absorption peaks related to OH^−^, H^+^, PO_4_^3−^, and CO_3_^2−^ of the three groups had almost no difference in position and intensity compared with fresh bone. It showed that the defatting treatments of acetone, gradient alcohol and high pressure washing could maintain the basic composition and natural structural state of collagen and inorganic matter. We also observed the efficacy of different defatting treatments on the DNA content, and the results showed that there was no significant difference in the DNA content of the three groups, demonstrating that high pressure washing, gradient alcohol, and acetone had the same ability to remove cells in bone. To observe whether the three types of defatting treatment would reduce the biomechanical properties of bone, we performed biomechanical tests on the three groups and the fresh group, mainly to observe the changes in their maximum stress and elastic modulus. It was found that there was no significant difference in maximum stress and elastic modulus between the three groups and the fresh group. Therefore, we believed that high pressure washing and gradient alcohol were effective treatments, which could achieve the same defatting efficacy as the acetone treatment, while ensuring the integrity of the internal structure of the bone and stable biomechanical properties.

### *Conclusion*

The results of this experiment indicate that the defatting efficiency was satisfactory at a time of 10 s under high pressure washing, and prolonging the time did not improve the defatting efficiency but reduced the biomechanical properties of the bone. In terms of defatting efficiency and its effect on biomechanical properties of bone, high pressure washing and gradient alcohol were similar to conventional acetone solvent extraction defatting. Most of the organic solvents used for defatting were toxic, and the residue in the material was not conducive to the adhesion of bone cells and the repair of bone tissue. Therefore, the selection of non‐toxic defatting treatment is of great significance for the antigen treatment of allogeneic bone. High pressure washing and gradient alcohol have the above characteristics, which lays a good foundation for the next step in constructing allograft bone for transplantation. However, these two treatments still require further *in vitro* cell tests, *in vivo* implantation experiments, and animal experiments to verify their safety and efficacy.
